# Management of Symptomatic Genu Recurvatum: Biomechanical Rationale, Etiology-Based Treatment Algorithm, and a Surgical Technical Blueprint

**DOI:** 10.3390/jcm15208006

**Published:** 2026-10-15

**Authors:** Vladan Stevanovic, Filip Cucakovic

**Affiliations:** 1 Institute for Orthopedics “Banjica”, 11040 Belgrade, Serbia; filip.cucakovic@gmail.com; 2 Faculty of Medicine, University of Belgrade, 11000 Belgrade, Serbia

**Keywords:** genu recurvatum, posterior tibial slope, proximal tibial osteotomy, sagittal alignment, knee instability, osteotomy, surgical technique

## Abstract

Symptomatic genu recurvatum is an uncommon sagittal-plane knee deformity associated with pathological hyperextension, pain, instability, gait disturbance, and functional limitation. Its heterogeneous causes and limited clinical evidence complicate treatment selection. This narrative review summarizes current concepts in biomechanics, etiologic classification, diagnostic evaluation, and management, with emphasis on posterior tibial slope (PTS) and slope-correcting proximal tibial osteotomy. Clinically relevant literature on genu recurvatum, sagittal knee alignment, osteotomy, and ligamentous instability was integrated with an illustrative previously reported case to provide a surgical technical blueprint. The available evidence supports an etiology-based approach. Reduced or reversed PTS is an important osseous contributor to pathological hyperextension, whereas soft-tissue, neuromuscular, and combined deformities require different strategies. In selected patients with osseous recurvatum and intact ligamentous restraints, anterior opening-wedge proximal tibial osteotomy can restore posterior slope and sagittal alignment. Critical technical steps include reproducible radiographic planning, fluoroscopically controlled K-wire placement, progressive posterior osteotomy completion, preservation of the posterior cortical hinge, gradual wedge opening, and stable fixation. An etiology-based diagnostic and treatment algorithm may facilitate individualized decision-making while avoiding unnecessary or incomplete procedures.

## 1. Introduction

Genu recurvatum is an uncommon sagittal plane deformity characterized by pathological knee hyperextension. Although small degrees of hyperextension may occur in asymptomatic individuals, symptomatic genu recurvatum is commonly defined in the contemporary literature as symptomatic hyperextension exceeding 5 degrees and may be associated with pain, instability, quadriceps fatigue, gait dysfunction, and functional impairment [1,2].

The pathomechanics of genu recurvatum are heterogeneous and may involve osseous deformity, soft-tissue insufficiency, neuromuscular dysfunction, or combined mechanisms. Dejour and colleagues emphasized the importance of distinguishing pure osseous recurvatum, soft-tissue recurvatum, and combined deformity, because treatment selection depends on identification of the dominant pathological mechanism [3].

Historically, treatment options included femoral flexion osteotomy, soft-tissue procedures, and proximal tibial osteotomy. Lecuire and colleagues provided one of the earliest focused descriptions of operative treatment, while later adult and pediatric series demonstrated that correction of tibial morphology could improve alignment and gait in selected patients [4,5,6].

In contemporary practice, posterior tibial slope has become a central biomechanical concept in sagittal knee stability. Decreased or reversed posterior tibial slope may shift tibiofemoral contact mechanics and facilitate pathological hyperextension. Therefore, restoration of physiological sagittal alignment has become a principal objective in appropriately selected patients with osseous genu recurvatum [2,7,8,9,10].

The purpose of this review is to summarize current concepts in the diagnosis and management of symptomatic genu recurvatum and to provide a case-based technical guide for anterior opening-wedge proximal tibial osteotomy. The manuscript is structured as a contemporary review with an illustrative technical case rather than as an isolated case report.

The authors have previously reported the clinical presentation and surgical management of a patient with unilateral idiopathic genu recurvatum in a regional peer-reviewed publication. The present manuscript substantially expands upon that preliminary report through a comprehensive literature review, updated biomechanical analysis, treatment algorithm, and detailed discussion of contemporary evidence [11].

## 2. Biomechanics of Genu Recurvatum

### 2.1. Sagittal Plane Alignment

Normal knee function depends on a complex interaction between osseous geometry, capsuloligamentous restraints, muscular control, and dynamic loading during gait. While coronal plane deformities have traditionally received greater attention, sagittal alignment is equally important for knee stability and physiological loading [1,2,3].

Genu recurvatum creates an abnormal extension moment during the stance phase. The ground reaction force may pass anterior to the knee center, exaggerating hyperextension and increasing stress on posterior capsular structures, the posterior cruciate ligament, posterolateral structures, and the articular surfaces [1,12,13,14].

### 2.2. Posterior Tibial Slope

Posterior tibial slope is one of the most important osseous determinants of sagittal knee stability. It is commonly measured as the angle between the tibial plateau and a line perpendicular to the anatomical axis of the tibia on a true lateral radiograph. Physiological values vary, but a posterior inclination of approximately 7 to 10 degrees is frequently considered normal [2,7,10,15,16].

Reduction in or reversal of posterior tibial slope promotes a recurvatum tendency by altering femorotibial contact and the relationship between the femur and tibia during weight-bearing. Conversely, increasing posterior tibial slope can reduce pathological hyperextension in osseous recurvatum, but excessive slope may increase anterior cruciate ligament loading [15,16,17,18].

**Figure 1:**
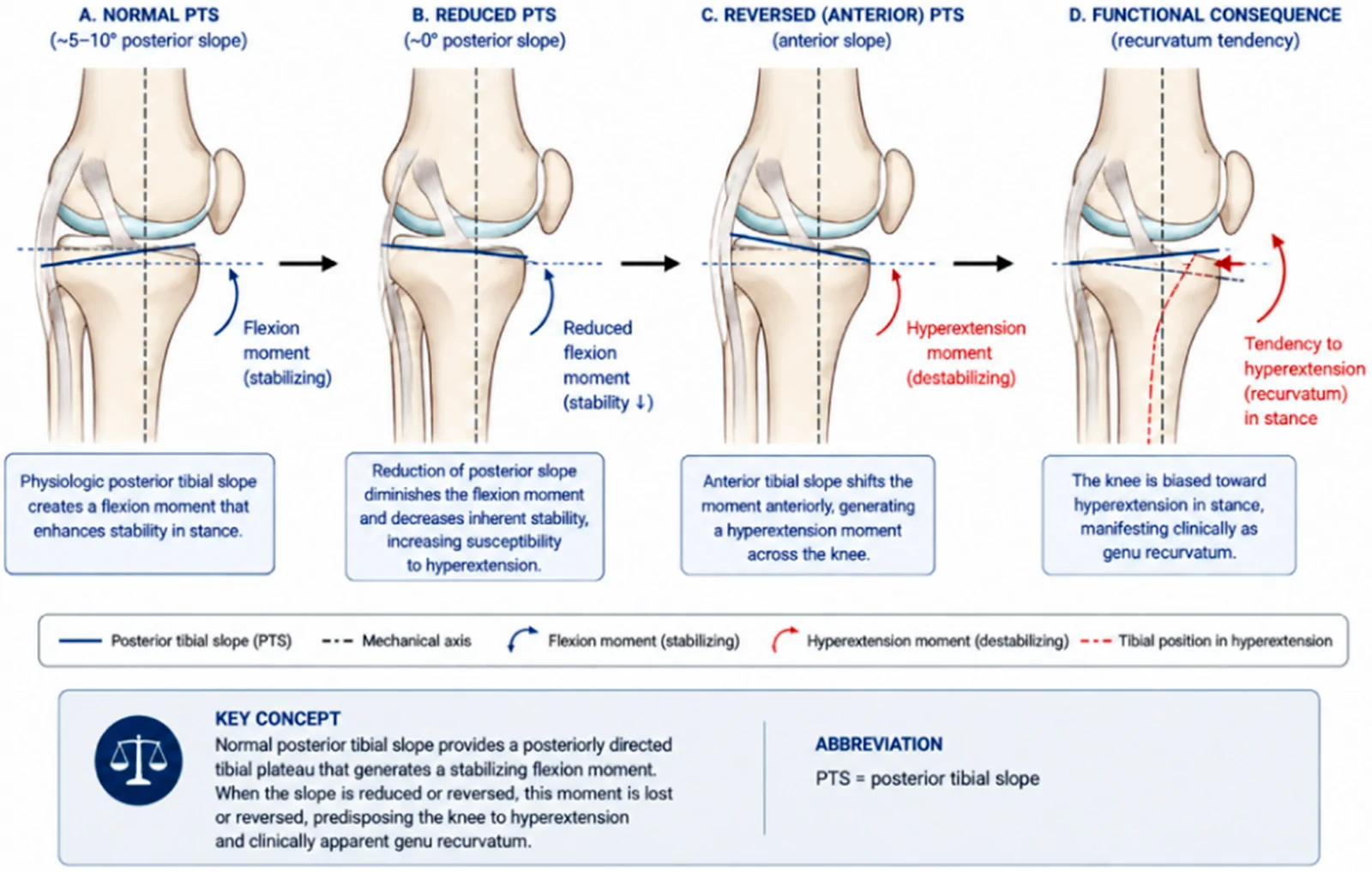
Biomechanical impact of posterior tibial slope (PTS) on sagittal knee balance and the tendency to genu recurvatum. Progressive loss of physiologic posterior slope, from physiologic through reduced and neutral PTS to reversed (anterior) slope, decreases the stabilizing sagittal geometry and increases the osseous predisposition to hyperextension.

The relationship between posterior tibial slope, femorotibial contact mechanics, and knee hyperextension is illustrated in Figure 1.

### 2.3. Influence on Cruciate Ligament and Posterolateral Structures

Changes in tibial slope modify cruciate ligament loading. Increased posterior tibial slope increases anterior tibial translation and anterior cruciate ligament graft force, while decreased or reversed slope may increase posterior translation tendency and hyperextension mechanics [15,16,17,18].

Posterior capsular structures, the oblique popliteal ligament, the posterior cruciate ligament, and the posterolateral corner contribute to resistance against hyperextension. Biomechanical work has demonstrated the importance of posterior soft-tissue structures in preventing knee hyperextension, particularly in combined instability patterns [12,13,14,19].

### 2.4. Gait Alterations

Patients with symptomatic genu recurvatum frequently develop gait abnormalities, including excessive stance-phase hyperextension, reduced shock absorption, shortened stride length, and compensatory hip or pelvic motion. In neuromuscular conditions, hyperextension may initially serve as a compensatory strategy for quadriceps weakness but may eventually lead to progressive posterior soft-tissue stretching and joint overload [1,20,21].

### 2.5. Biomechanical Rationale for Correction

The biomechanical goal of surgical correction is restoration of physiological sagittal alignment while preserving or restoring knee stability. In osseous deformities, anterior opening-wedge proximal tibial osteotomy increases posterior tibial slope, shifts the femorotibial contact relationship, reduces pathological extension forces, and may improve dynamic stability [2,7,8,9,10,22,23,24,25,26,27].

## 3. Etiology and Classification

Symptomatic genu recurvatum may be congenital, developmental, post-traumatic, neuromuscular, ligamentous, or idiopathic. Although the final clinical manifestation is excessive knee hyperextension, the mechanism producing the deformity differs substantially among patients. Accurate etiological diagnosis is essential because the optimal treatment for osseous deformity differs from that for soft-tissue insufficiency or neuromuscular imbalance [1,2,3].

### 3.1. Congenital and Developmental Causes

Congenital genu recurvatum is rare and may occur as an isolated abnormality or in association with syndromic conditions and generalized laxity. Developmental deformity may also occur after partial growth arrest, particularly involving the anterior proximal tibial physis, resulting in progressive reduction in posterior tibial slope during growth [28,29].

### 3.2. Post-Traumatic Causes

Post-traumatic genu recurvatum may follow proximal tibial fractures, physeal injuries, malunion, complex knee trauma, or previous osteotomy. These deformities may be purely osseous or combined with ligamentous injury. The distinction is important because isolated osteotomy may not be sufficient in combined or multiplanar instability [22,23,24,29,30,31,32,33].

### 3.3. Neuromuscular Causes

Neuromuscular disorders, including poliomyelitis, cerebral palsy, stroke, spinal cord injury, and peripheral nerve dysfunction, may produce dynamic recurvatum through muscle imbalance and impaired motor control. Historic surgical options included femoral flexion osteotomy and soft-tissue stabilization procedures [20,21,34,35].

### 3.4. Ligamentous and Soft-Tissue Causes

Isolated soft-tissue recurvatum may occur in the setting of posterior capsular laxity, posterior cruciate ligament deficiency, posterolateral corner insufficiency, generalized hypermobility, or chronic multiligament instability. In such cases, ligamentous reconstruction or capsular procedures may be indicated, either alone or combined with osteotomy when malalignment is also present [13,14,36,37,38].

### 3.5. Idiopathic Deformity

Idiopathic genu recurvatum remains a diagnosis of exclusion. Patients typically present with progressive symptomatic hyperextension without a clear history of trauma, neuromuscular disease, or major ligament injury. In this subgroup, subtle osseous abnormalities of the proximal tibia and altered posterior tibial slope may be clinically relevant [2,7].

The most clinically useful classification separates osseous, soft-tissue, and combined deformities. Table 1 summarizes common etiologies, pathomechanisms, and treatment strategies.

## 4. Diagnostic Evaluation

Accurate diagnosis requires integration of history, clinical examination, radiographs, advanced imaging, and functional assessment. The diagnostic work-up should define the magnitude of hyperextension, identify the primary etiology, quantify posterior tibial slope, assess ligament integrity, and determine whether the deformity is osseous, soft-tissue, neuromuscular, or combined [1,2,3].

### 4.1. Clinical Examination and Gait Assessment

Clinical evaluation should include assessment of passive and active hyperextension, limb alignment, gait, generalized ligamentous laxity, quadriceps and hamstring strength, neurological status, and signs of cruciate or posterolateral instability. For prone heel-height measurement, both lower limbs are positioned comparably, and the vertical difference between the heels is recorded during passive knee extension. This documents side-to-side extension asymmetry and should be interpreted together with goniometric assessment; the validation study primarily evaluated subtle flexion contractures after ACL reconstruction [39]. A separate heel-height test has been studied as an adjunct for detecting combined ACL and fibular collateral ligament injury [40], rather than as a standalone test for osseous recurvatum.

### 4.2. Radiographic Evaluation

Standing anteroposterior, true lateral, and long-leg alignment radiographs are recommended. The lateral radiograph is essential for posterior tibial slope (PTS) measurement, while long-leg films identify associated coronal malalignment that may influence osteotomy planning [7,8,22,41]. For reproducibility, preoperative and postoperative PTS should be measured using the same radiographic method on a true lateral image. In this manuscript, PTS is defined as the angle between the tibial plateau line and a line perpendicular to the anatomical axis of the proximal tibia. A posteriorly descending plateau is reported as a positive posterior slope, whereas reversal toward an anterior slope is reported as a negative value. Small differences between measurements may arise from radiographic projection; therefore, strict lateral positioning and consistent axis definition are essential when comparing preoperative and postoperative values.

### 4.3. MRI and CT Planning

Magnetic resonance imaging is useful for evaluating the anterior cruciate ligament, posterior cruciate ligament, menisci, cartilage, posterior capsule, and posterolateral corner. Computed tomography or multislice computed tomography may improve preoperative understanding of proximal tibial morphology, rotational alignment, osteotomy level, wedge size, and planned correction, particularly in complex or multiplanar deformity [22,23,24,25,29,32,33].

**Figure 2:**
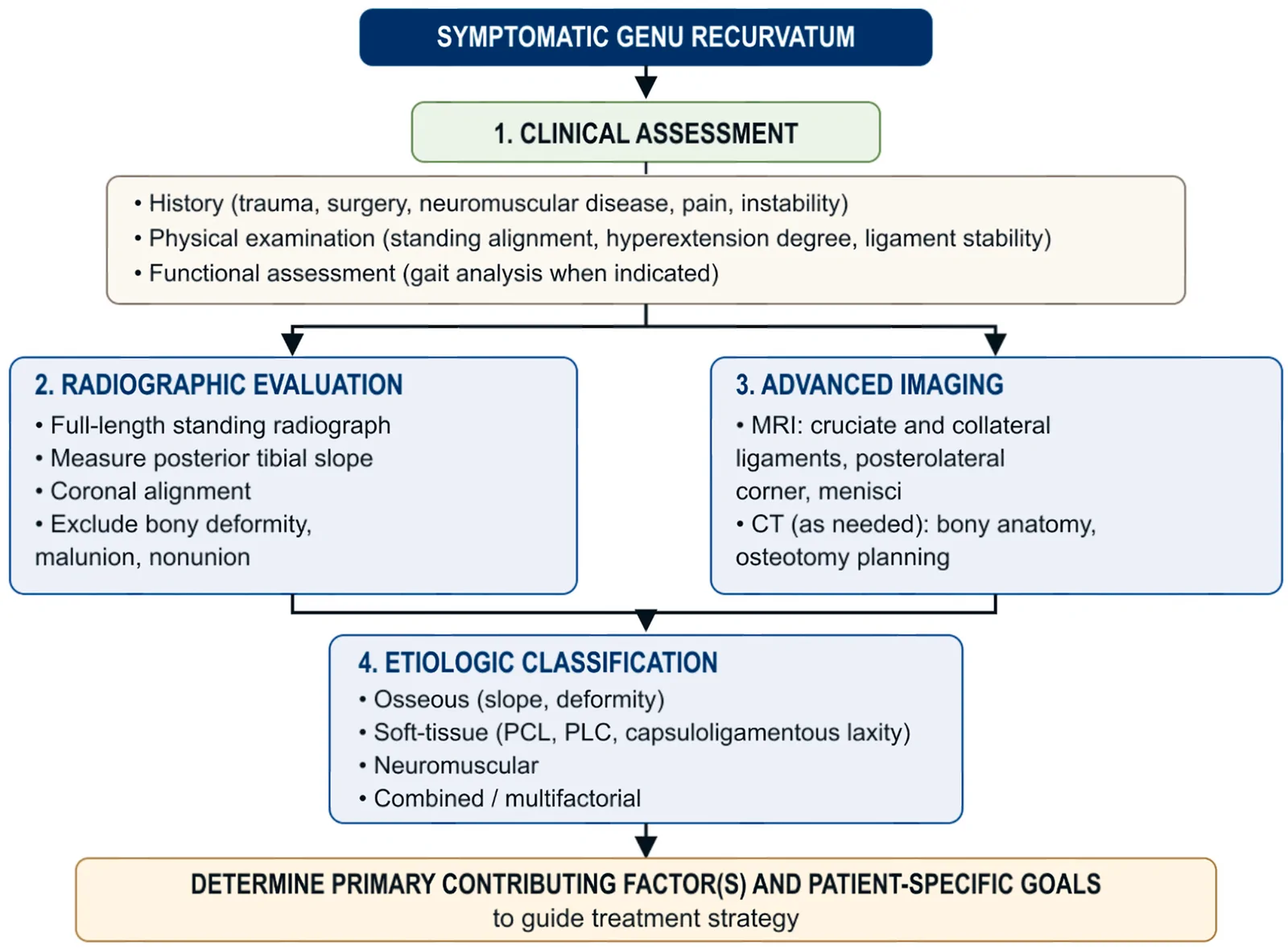
Diagnostic algorithm for evaluation and treatment planning in patients with symptomatic genu recurvatum. Clinical assessment is followed by radiographic evaluation and advanced imaging as indicated. Findings from both assessments inform etiologic classification, which precedes identification of the primary contributing factor(s) and patient-specific treatment goals.

A proposed diagnostic pathway is presented in Figure 2. Clinical assessment is followed by radiographic analysis and posterior tibial slope measurement. Advanced imaging is then used to distinguish osseous from soft-tissue pathology and to guide individualized treatment selection.

## 5. Surgical Indications and Treatment Principles

Management should be individualized according to etiology, deformity magnitude, functional limitation, patient age, activity level, and associated instability. Nonoperative treatment may be appropriate in mild or minimally symptomatic cases, especially when dynamic neuromuscular mechanisms predominate. Physiotherapy, strengthening, gait retraining, activity modification, orthoses, and functional bracing may improve symptoms but rarely correct established osseous deformity [1,20,21].

Surgical intervention should be considered when painful hyperextension, subjective instability, progressive deformity, gait dysfunction, or activity limitation persists despite conservative treatment and corresponds to objective structural pathology. Such pathology includes abnormal posterior tibial slope, other osseous malalignment, demonstrable capsuloligamentous insufficiency, or a combination of malalignment and ligament insufficiency. Symptoms and functional limitation therefore support the indication for surgery, while the structural findings determine the corrective strategy [2,3,7,8,22].

Treatment selection should be etiology-based. Patients with osseous recurvatum and decreased or reversed posterior tibial slope are generally candidates for corrective osteotomy, whereas patients with predominant soft-tissue insufficiency may require ligament reconstruction or capsular procedures. Combined deformities may require combined correction [30,31,36,37,38].

Table 2 summarizes treatment selection according to etiology.

## 6. Surgical Options

### 6.1. Proximal Tibial Osteotomy

Proximal tibial osteotomy remains the most commonly reported surgical procedure for osseous genu recurvatum. The aim is restoration of posterior tibial slope and correction of sagittal malalignment [2,5,7,8,9,10,22,26,27].

### 6.2. Anterior Opening-Wedge Proximal Tibial Osteotomy

Anterior opening-wedge proximal tibial osteotomy is one of the most commonly described slope-correcting procedures for symptomatic genu recurvatum associated with reduced or reversed posterior tibial slope. The procedure creates an anterior opening wedge while preserving the posterior cortical hinge. Controlled opening increases posterior tibial slope and reduces pathological hyperextension [7,8,9,10,22,23,24,25,26,27].

### 6.3. Closing-Wedge and Anterior Displacement Osteotomies

Closing-wedge or anterior displacement osteotomies have been described, particularly in pediatric or paralytic deformities. Advantages include broad cancellous bone contact and potentially reliable union, while disadvantages include shortening, limited fine-tuning, and technical complexity [20,34,42].

### 6.4. Gradual Correction with External Fixation

Ilizarov-based correction allows gradual adaptation of soft tissues, multiplanar correction, and limb-length adjustment. It is particularly useful in severe or complex deformity but is associated with longer treatment duration, pin-tract morbidity, and substantial patient burden [6,43].

### 6.5. Soft-Tissue and Combined Procedures

Patients with ligamentous recurvatum may require posterior capsular procedures, posterior cruciate ligament reconstruction, posterolateral corner reconstruction, or multiligament reconstruction. When malalignment and ligament deficiency coexist, osteotomy may be required to protect ligament reconstruction and restore mechanical balance [13,14,30,31,36,37,38].

Reported complications differ by technique and are incompletely documented. In the 30-patient tuberosity-sparing anterior opening-wedge series, symptomatic hardware required removal in 17 patients (56.7%), and one patient (3.3%) developed an operative-site infection [9]. After conventional anterior opening-wedge osteotomy, Van Raaij and de Waal Malefijt reported patella infera associated with patellofemoral complaints, persistent iliac-crest donor-site pain, and lateral thigh dysesthesia; one patient sustained an ACL injury during sport two years after surgery [7]. Kim et al. reported no complications in five patients treated with anterior open-wedge oblique osteotomy, but this small series cannot establish a low complication rate [8]. Choi et al. reported complications in three of ten patients undergoing Ilizarov correction, comprising patella infera, pin-track infection, and transient peroneal nerve palsy [6]. Capsular procedures may be followed by recurrent laxity or loss of extension from excessive tightening [38]. Technique-specific complication estimates for closing-wedge/anterior displacement and combined procedures remain insufficient for reliable comparison; absence of a reported event should not be interpreted as absence of risk.

Table 3 compares major surgical options and their advantages and limitations.

## 7. Narrative Review of the Clinical Literature

Review approach: This article was designed as a narrative review rather than a systematic review. The literature synthesis prioritizes clinically relevant peer-reviewed reports addressing symptomatic genu recurvatum, posterior tibial slope, sagittal knee biomechanics, proximal tibial osteotomy, and associated ligamentous instability. The purpose was to integrate biomechanical rationale with practical diagnostic and surgical decision-making; no claim of exhaustive systematic retrieval or meta-analysis is made. The literature identified with AI-assisted search support was treated only as candidate material; bibliographic details and scientific relevance were checked against the original journal or PubMed records before inclusion. Table S1 contains 24 publications: 8 clinical/case series, 2 case reports, 2 clinical reports, 2 historical reports, 5 technical notes, 1 technical report, 1 technical review, 1 systematic review, 1 narrative/technical review, and 1 narrative review. These counts follow the design labels in Table S1; the 8 clinical/case series comprise 6 entries labelled as clinical series and 2 labelled as case series.

The literature on symptomatic genu recurvatum remains limited and consists predominantly of historical descriptions, technical notes, case reports, and retrospective series. In the 2020 systematic review by Dean et al. [2], only six clinical studies comprising 80 patients met the inclusion criteria. Since that review, additional technical reports and a 30-patient case series have expanded the evidence base, but high-level comparative evidence remains absent [9,26,27,33].

Historical reports established several operative principles. Early surgical approaches focused on paralytic recurvatum and soft-tissue procedures, including femoral flexion osteotomy and triple tenodesis. Later studies introduced proximal tibial osteotomy and gradual correction for osseous deformities [4,5,6,20,21,34,35].

Contemporary literature increasingly supports slope-correcting osteotomy for appropriately selected patients with symptomatic genu recurvatum. Van Raaij and de Waal Malefijt reported symptomatic improvement after anterior opening-wedge osteotomy in idiopathic hyperextension knees; Kim and colleagues described an anterior open-wedge oblique technique; and subsequent technical reports further refined slope-correcting osteotomy for posterior instability and recurvatum [7,8,23,25,26,33]. Most importantly, Chou et al. reported a 30-patient series in which tuberosity-sparing anterior opening-wedge tibial osteotomy significantly improved recurvatum angle and all KOOS domains while preserving coronal alignment and patellar height [9].

For traumatic or complex deformities, flexion osteotomy and combined correction strategies have been described. Case-based and deformity-analysis literature emphasizes individualized planning, particularly when coronal malalignment, osteochondral pathology, or ligament deficiency coexist [22,24,25,30,31,32,33].

Two published examples illustrate alternative planning requirements. Chen et al. [29] described a 16-year-old patient with 45 degrees of recurvatum and 4 cm of limb shortening after a neglected proximal tibial physeal injury. Opening-wedge osteotomy with allograft reconstruction and dual buttress plates produced equal limb lengths and a painless range of motion of 0 to 140 degrees at three years. Reddy et al. [33] described symptomatic recurvatum after failed high tibial osteotomy and PCL reconstruction, with reversed tibial slope and varus malalignment. Their anterior open-wedge technique addressed the sagittal and coronal deformities simultaneously, illustrating the need to plan both planes when previous osteotomy and ligament failure coexist. These are literature-derived examples, distinct from the authors’ illustrative case.

Across published studies, correction generally improves hyperextension, restores sagittal alignment, and may improve pain, subjective stability, and function [2,9,27]. However, direct comparison among studies remains limited by heterogeneous etiologies, inconsistent outcome reporting, variable definitions of recurvatum, and predominantly low-level evidence [2,10].

Representative studies are summarized in Table S1.

## 8. Illustrative Case and Surgical Technical Blueprint

### 8.1. Patient Presentation

A 32-year-old male patient presented with progressive unilateral left knee hyperextension, subjective instability, and pain during high-demand physical activity [11]. Symptoms progressed over three years without known major trauma. Clinical examination demonstrated approximately 15 degrees of hyperextension, preserved range of motion, clinically stable cruciate ligaments, intact skin, and normal distal neurovascular status. The clinical presentation and initial case description have been reported previously; the present manuscript uses the case solely as an illustrative framework for a substantially expanded biomechanical, diagnostic, and technical discussion. The clinical deformity is shown in Figures S1 and S2.

### 8.2. Preoperative Evaluation and Planning

Radiographic evaluation demonstrated abnormal sagittal alignment with a reduced positive posterior tibial slope of approximately +4.4 degrees, calculated from the annotated plateau–tibial-axis angle of 94°24′46″ in Figure S3 by subtracting 90 degrees. This is an interpretation of the existing radiographic annotation, not a new measurement of the original radiograph. Magnetic resonance imaging and arthroscopic assessment confirmed intact anterior and posterior cruciate ligaments. Multislice computed tomography was used for preoperative planning. The preoperative slope measurement is shown in Figure S3.

Because the dominant pathology was osseous, the cruciate ligaments were intact, and the abnormal sagittal morphology was characterized by reduced positive PTS, isolated anterior opening-wedge proximal tibial osteotomy was selected. The therapeutic objective was not simply correction of the visible hyperextension but restoration of physiological sagittal osseous geometry while avoiding unnecessary ligament reconstruction.

### 8.3. Operative Technique

Step 1. Surgical setup and K-wire orientation

Surgical setup and osteotomy orientation: Diagnostic arthroscopy may first be performed when intra-articular pathology or ligament integrity requires confirmation. The proximal tibia is then exposed through a longitudinal anterior approach. Two parallel K-wires are inserted approximately 5 mm distal to the tibial tuberosity and directed posteriorly under fluoroscopic guidance. The wires define the intended osteotomy plane and serve as a visual and radiographic reference during bone cutting. Their trajectory should be verified in orthogonal fluoroscopic projections before the osteotomy is initiated.

Step 2. Osteotomy completion and posterior hinge protection

Posterior osteotomy completion and hinge protection: The anterior and central portions of the osteotomy are developed with a saw and completed progressively with osteotomes. Particular attention is paid to the posterior cortex, which functions as the principal stabilizing hinge during slope correction. The osteotomy is advanced from anterior to posterior in a controlled fashion with repeated fluoroscopic verification rather than being completed blindly. A posterior cortical bridge is deliberately preserved. Forceful wedge opening before adequate osteotomy completion is avoided because stress concentration at the posterior cortex may propagate an unintended hinge fracture, destabilize the construct, and compromise control of the planned correction.

Controlled opening and correction: After satisfactory osteotomy completion has been confirmed fluoroscopically, the anterior wedge is opened gradually and symmetrically. Opening is performed in small increments while the posterior hinge is repeatedly assessed fluoroscopically for continuity, and the sagittal correction is monitored. The correction is stopped when the preoperatively planned slope and satisfactory clinical elimination of pathological hyperextension are achieved. Overcorrection should be avoided because excessive posterior tibial slope may adversely alter cruciate-ligament biomechanics. In the illustrative case, approximately +11 degrees is the final postoperative PTS (Figure S7), not the magnitude of correction. Relative to the approximately +4.4-degree preoperative slope derived from Figure S3, the implied increase is approximately 6.6 degrees. The final slope is about 1 degree above the 7–10-degree range cited in Section 2.2 and therefore represents a small radiographic overcorrection relative to that range; it should not be described unconditionally as physiological. Although the reported clinical outcome was favorable, this single observation does not establish an optimal target or exclude longer-term effects of increased ACL loading [15].

Graft insertion and fixation. Once the desired correction has been confirmed, the osteotomy gap is supported with structural allograft. Definitive fixation is obtained with an anteromedial TomoFix locking plate. Final fluoroscopic assessment confirms maintenance of the correction, implant position, preservation of the posterior hinge, and absence of unintended propagation of the osteotomy.

### 8.4. Technical Checkpoint

Before wound closure, the surgeon should confirm four points: preservation of the posterior cortical hinge, achievement of the planned sagittal correction, stable graft-supported fixation, and satisfactory implant position on final fluoroscopic views.

### 8.5. Postoperative Rehabilitation and Outcome

Early postoperative management included pain control, edema reduction, quadriceps activation, and range-of-motion exercises. Weight-bearing was restricted during the early healing phase and progressively increased after radiographic evidence of union. Bone union was achieved by three months. At follow-up, the patient walked independently without complaints, with normal range of motion, restored sagittal alignment, and no residual instability. Postoperative clinical and radiographic findings are shown in Figures S6 and S7.

## 9. Discussion

A central concept emerging from the available literature is that restoration of appropriate sagittal osseous geometry is fundamental in the management of symptomatic osseous genu recurvatum. Although the evidence base remains limited, published reports and the recent 30-patient clinical series suggest that correction of abnormal sagittal alignment can reduce pathological hyperextension and improve pain and function in selected patients [2,7,8,9,23,24,25,27].

The understanding of genu recurvatum has evolved from a purely clinical description of hyperextension toward a biomechanically oriented model based on sagittal plane alignment. Dejour and colleagues provided an important conceptual framework by separating osseous, soft-tissue, and combined deformities. This classification remains clinically valuable because treatment should address the primary pathology rather than the appearance of hyperextension alone [3].

Posterior tibial slope is a major modifiable osseous parameter influencing sagittal knee stability. Biomechanical studies demonstrate that modification of tibial slope alters cruciate ligament forces and knee kinematics. While increased posterior slope may increase anterior cruciate ligament loading, decreased or reversed slope promotes posterior translation and hyperextension mechanics. These concepts explain the rationale for slope-increasing osteotomy in osseous recurvatum and PCL-related posterior instability [10,15,16,17,18].

The illustrative case supports this concept. The patient had unilateral symptomatic hyperextension, intact cruciate ligaments, and abnormal posterior tibial slope. Correction through anterior opening-wedge proximal tibial osteotomy restored sagittal alignment and resolved functional instability. This outcome is consistent with published anterior opening-wedge, oblique, and flexion-osteotomy reports [7,8,9,23,25,26,27].

The main advantage of anterior opening-wedge proximal tibial osteotomy is direct and adjustable restoration of posterior tibial slope while preserving limb length. Stable locking-plate fixation and graft support allow maintenance of correction. The critical technical vulnerability is the posterior cortical hinge. An unintended posterior hinge fracture may convert a controlled opening-wedge correction into an unstable osteotomy, impair fine adjustment of sagittal alignment, and increase the risk of correction loss. For this reason, the posterior osteotomy should be completed progressively under fluoroscopic control, a cortical bridge should be preserved, and wedge opening should proceed gradually with repeated hinge assessment. Precise planning is equally important because undercorrection may result in persistent hyperextension, whereas overcorrection may alter cruciate-ligament biomechanics [8,15,23,25].

Soft-tissue and combined deformities require different strategies. Chronic posterior cruciate ligament deficiency, posterolateral corner insufficiency, or multiligament instability may require reconstruction. In malaligned knees, osteotomy may be necessary to protect ligament reconstruction and restore mechanical balance [13,14,30,31,36,37,38].

Several limitations of the current evidence should be acknowledged. Although the evidence base has expanded with a recent 30-patient series and a contemporary narrative review, most published data remain retrospective or technical, etiologies are heterogeneous, outcome measures are inconsistent, and long-term comparative data are limited [9,10]. There is no universal target for postoperative posterior tibial slope. Consequently, individualized deformity analysis remains essential, and multicenter prospective studies are needed.

A clinically useful contribution of the present review is the integration of biomechanical principles, etiologic classification, practical surgical decision-making, a technical blueprint, and a treatment algorithm within a single framework. Rather than proposing a universal operative solution, the algorithm emphasizes identification of the dominant pathological mechanism and individualized treatment planning.

## 10. Clinical Pearls and Pitfalls

Clinical pearls include careful etiological diagnosis, reproducible posterior tibial slope measurement, CT-based planning when required, fluoroscopically controlled K-wire placement, progressive posterior osteotomy completion, deliberate preservation of the posterior cortical hinge, gradual wedge opening, fluoroscopic verification of correction, stable locking-plate fixation, and structured rehabilitation. Common pitfalls include incomplete deformity analysis, inconsistent radiographic measurement, premature or forceful wedge opening, posterior hinge fracture, undercorrection, overcorrection, failure to identify associated ligamentous pathology, inadequate fixation or graft support, and poorly structured rehabilitation. Table S2 summarizes the major clinical pearls, their technical rationale, and the consequences of common errors.

## 11. Proposed Treatment Algorithm

**Figure 3:**
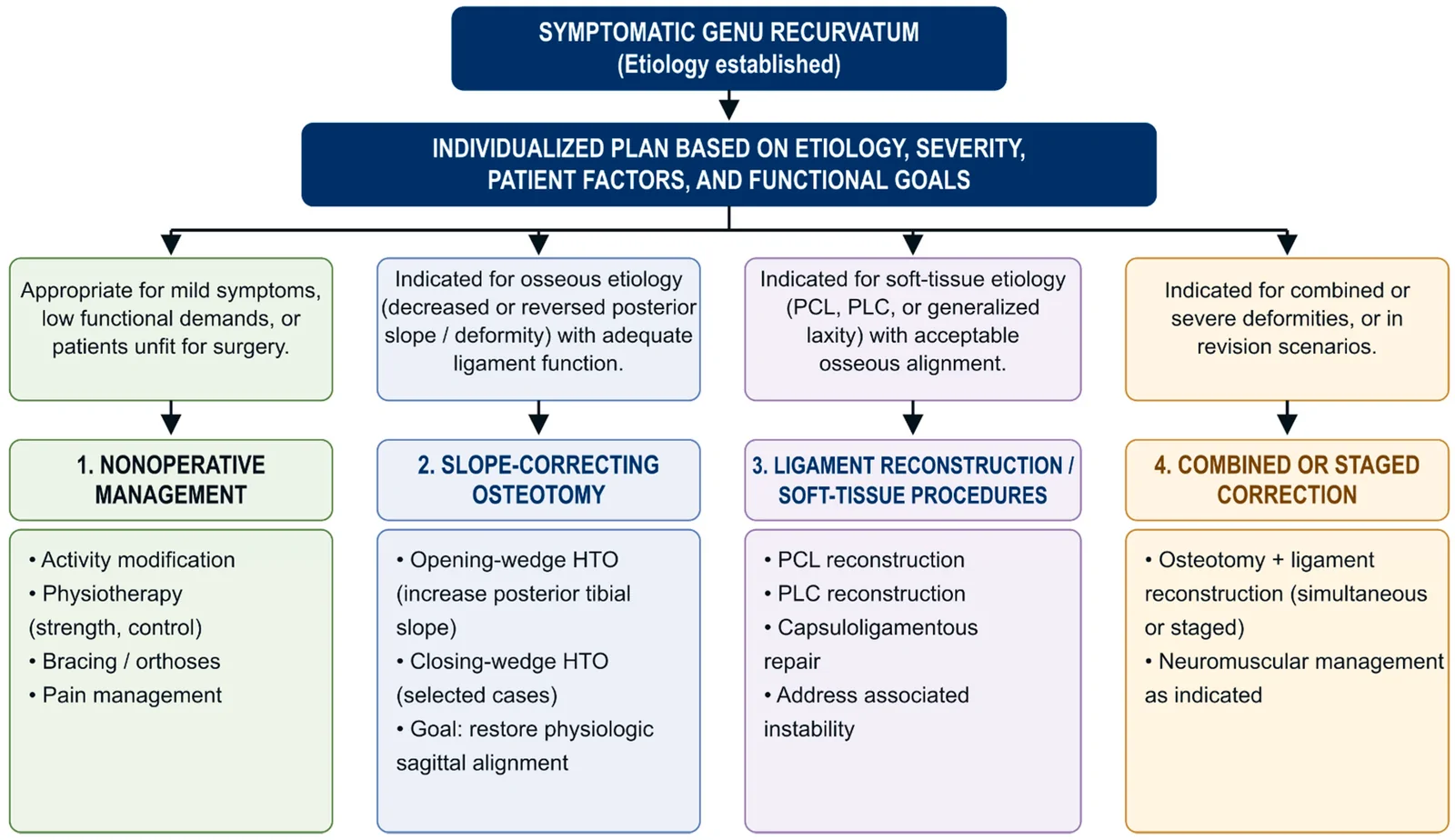
Proposed treatment algorithm for symptomatic genu recurvatum. The pathway begins with confirmation and quantification of symptomatic hyperextension, followed by standardized posterior tibial slope analysis and assessment of ligament integrity. An individualized plan based on etiology, severity, patient factors, and functional goals is established before the treatment branches. The indications then guide the corresponding options: nonoperative management for mild functional deformity, slope-correcting osteotomy for osseous recurvatum with abnormal PTS, soft-tissue reconstruction for ligamentous insufficiency, and combined or staged correction for mixed deformity.

The proposed treatment algorithm begins with confirmation of symptomatic hyperextension and quantification of deformity. Posterior tibial slope measurement and MRI assessment of ligamentous structures are then performed. CT-based deformity analysis is recommended when surgical correction is considered. Patients with mild symptoms may undergo rehabilitation and bracing; those with osseous deformity and abnormal slope are candidates for osteotomy; those with predominant soft-tissue insufficiency may require reconstruction; and combined deformities may require combined procedures. The algorithm is illustrated in Figure 3.

## 12. Limitations

The current evidence regarding symptomatic genu recurvatum remains limited and is largely based on retrospective case series, technical notes, and case reports. The illustrative case included in this manuscript represents a single-patient experience and should therefore be interpreted cautiously. Furthermore, outcome measures and definitions of pathological hyperextension vary substantially across published studies. Despite these limitations, the present review integrates available biomechanical concepts, clinical evidence, and surgical strategies into a practical framework for diagnosis and treatment. Future multicenter prospective studies are required to better define optimal indications, correction targets, and long-term outcomes.

## 13. Conclusions

Symptomatic genu recurvatum is a challenging sagittal plane deformity with heterogeneous etiologies. Contemporary evidence supports an etiology-based approach in which posterior tibial slope analysis plays a central role. In appropriately selected patients with symptomatic osseous recurvatum and reduced or reversed posterior tibial slope, anterior opening-wedge proximal tibial osteotomy has been associated with correction of sagittal alignment, reduction in pathological hyperextension, and improvement in functional outcomes. Because current evidence remains limited, individualized deformity analysis is essential, and future multicenter prospective studies are needed to define optimal indications, correction targets, and long-term outcomes.

## Figures and Tables

**Table 1:** Etiology, pathomechanism, and preferred treatment strategy.

Etiology	Primary Mechanism	Typical Findings	Preferred Treatment
Congenital	Developmental soft-tissue or osseous abnormality	Early-onset hyperextension, syndromic association possible	Casting, orthoses, physiotherapy, selective surgery
Growth arrest	Anterior proximal tibial physeal disturbance	Progressive reduction in posterior tibial slope	Corrective proximal tibial osteotomy
Post-traumatic	Fracture malunion or combined ligament injury	Sagittal or multiplanar deformity	Osteotomy with or without ligament reconstruction
Neuromuscular	Muscle imbalance and dynamic instability	Stance-phase hyperextension, weakness	Rehabilitation, bracing, muscle balancing, selective osteotomy
Ligamentous laxity	Posterior capsular, PCL, or posterolateral insufficiency	Hyperextension with instability	Ligament reconstruction or capsular procedure
Idiopathic	Unclear, frequently osseous slope abnormality	Progressive unilateral or bilateral hyperextension	Individualized correction based on slope and symptoms

**Table 2:** Etiology-based treatment selection.

Dominant Pathology	Key Diagnostic Features	Treatment Strategy
Mild functional deformity	Minimal symptoms, no progressive structural abnormality	Physiotherapy, bracing, activity modification
Osseous recurvatum	Reduced or reversed posterior tibial slope, intact ligaments	Slope-correcting proximal tibial osteotomy
Soft-tissue recurvatum	Capsuloligamentous laxity, PCL or posterolateral deficiency	Ligament reconstruction or capsular stabilization
Neuromuscular recurvatum	Muscle imbalance, dynamic hyperextension	Multidisciplinary rehabilitation, orthoses, selective surgery
Combined deformity	Malalignment with ligament insufficiency	Combined or staged osteotomy and reconstruction

**Table 3:** Comparative analysis of surgical techniques.

Technique	Primary Indication	Advantages	Limitations
Anterior opening-wedge proximal tibial osteotomy	Osseous recurvatum with reduced PTS	Direct slope correction, adjustable, preserves limb length	Requires grafting or spacer support; hinge fracture possible
Closing-wedge or anterior displacement osteotomy	Selected osseous or paralytic deformity	Bone contact and union potential	Shortening, less adjustability, technical complexity
Oblique opening-wedge osteotomy	Complex sagittal or multiplanar deformity	Allows combined correction	Demanding planning and execution
Ilizarov or circular external fixation	Severe or multiplanar deformity	Gradual correction, soft-tissue adaptation	Pin-tract complications, long treatment duration
Ligament or capsular reconstruction	Soft-tissue insufficiency	Restores passive restraint	Does not correct osseous pathology
Combined procedures	Mixed osseous and soft-tissue pathology	Comprehensive correction	Higher complexity and rehabilitation demand

## Data Availability

No new dataset was generated for this narrative review. Data relating to the illustrative clinical case are not publicly available because of patient privacy considerations.

## Supplementary Materials

The following supporting information is available at https://www.mdpi.com/article/10.3390/jcm15208006/s1: Figures S1–S7, clinical, radiographic, and intraoperative images from the illustrative case; Table S1, representative clinical and technical literature on symptomatic genu recurvatum; Table S2, clinical pearls and pitfalls. All references cited in the Supplementary Materials are included in the main reference list.
