# Supplementary material for: Management of Symptomatic Genu Recurvatum: Biomechanical Rationale, Etiology-Based Treatment Algorithm, and a Surgical Technical Blueprint
Source: J Clin Med. 2026 Oct 15;15(20):8006. doi: 10.3390/jcm15208006 (PMC13705637; doi:10.3390/jcm15208006)
Supplement: Supplementary file 1 [file jcm-15-08006-s001.zip › jcm-4561601-supplementary.pdf]

Supplementary Materials

Management of Symptomatic Genu Recurvatum: Biomechanical Rationale, Etiology-Based Treatment Algorithm, and a Surgical Technical Blueprint

The supplementary figures below correspond to the illustrative clinical case cited in the main manuscript. Figure numbering is independent of the main-text figures and begins with Figure S1.

Supplementary Figures

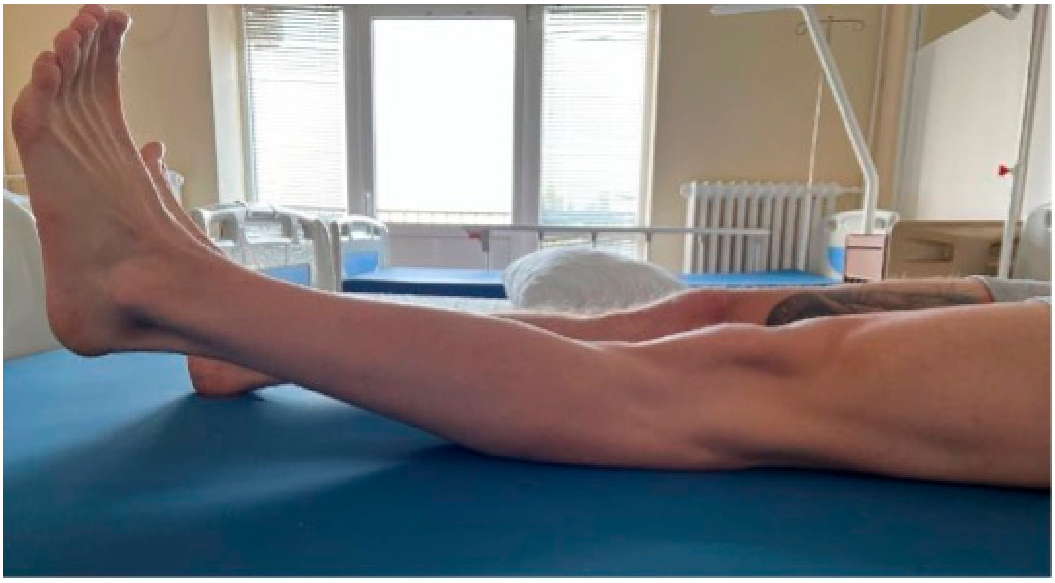

Figure S1. Preoperative clinical appearance demonstrating symptomatic left-sided genu recurvatum in the supine position.. Reprinted from Ref.[11]

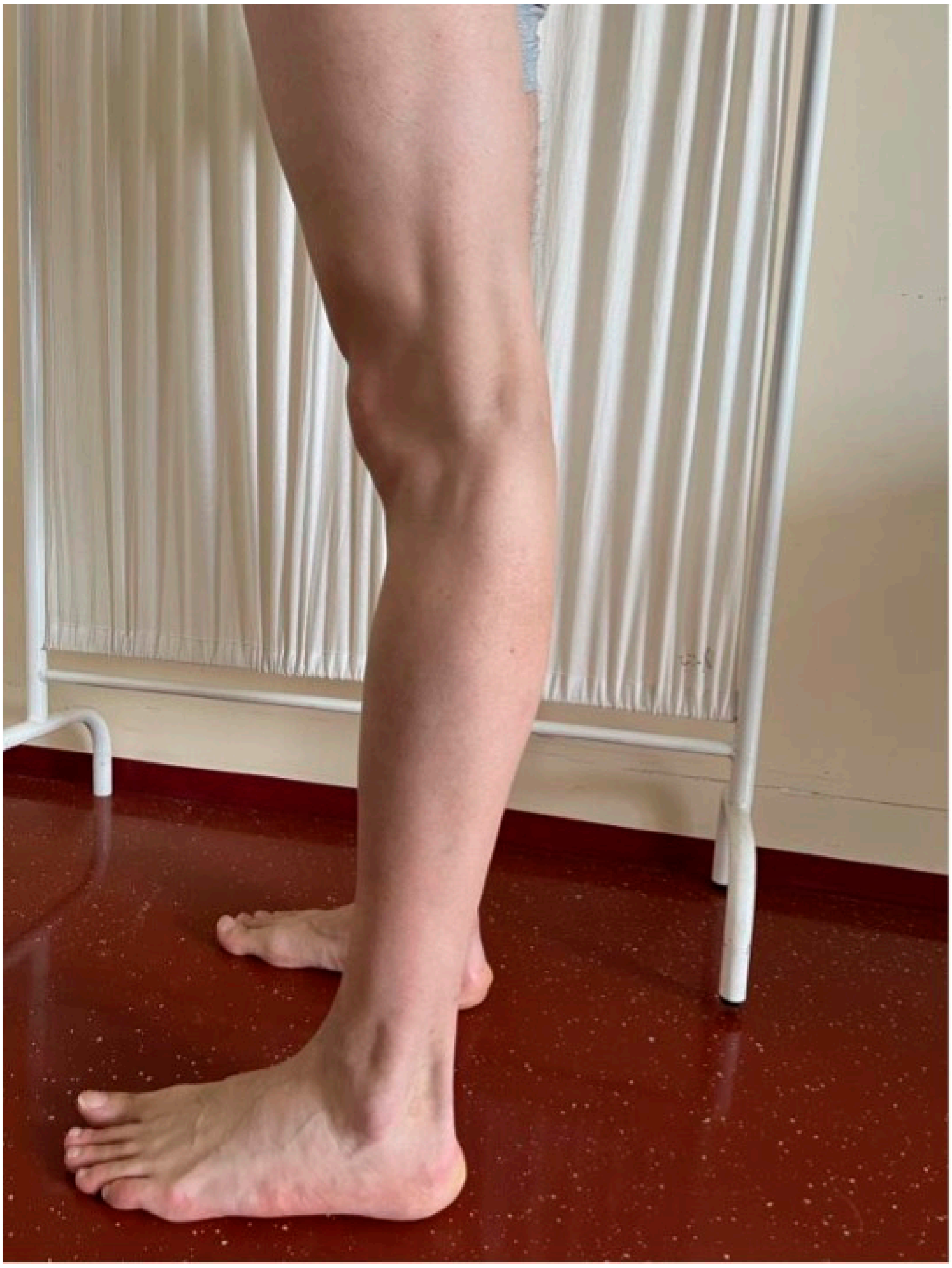

Figure S2. Standing clinical appearance demonstrating pathological hyperextension during weight-bearing.. Reprinted from Ref.[11]

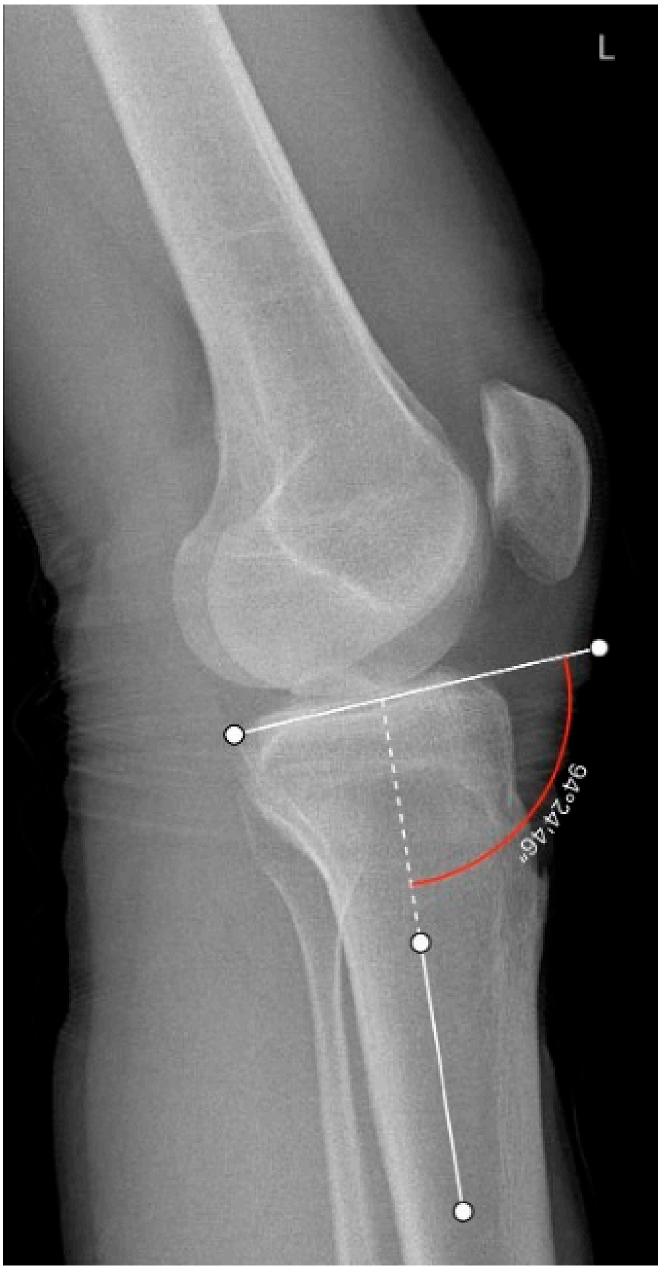

Figure S3. Preoperative lateral radiograph demonstrating reduced positive posterior tibial slope of approximately +4.4° and associated sagittal-plane deformity. Reprinted from Ref.[11]

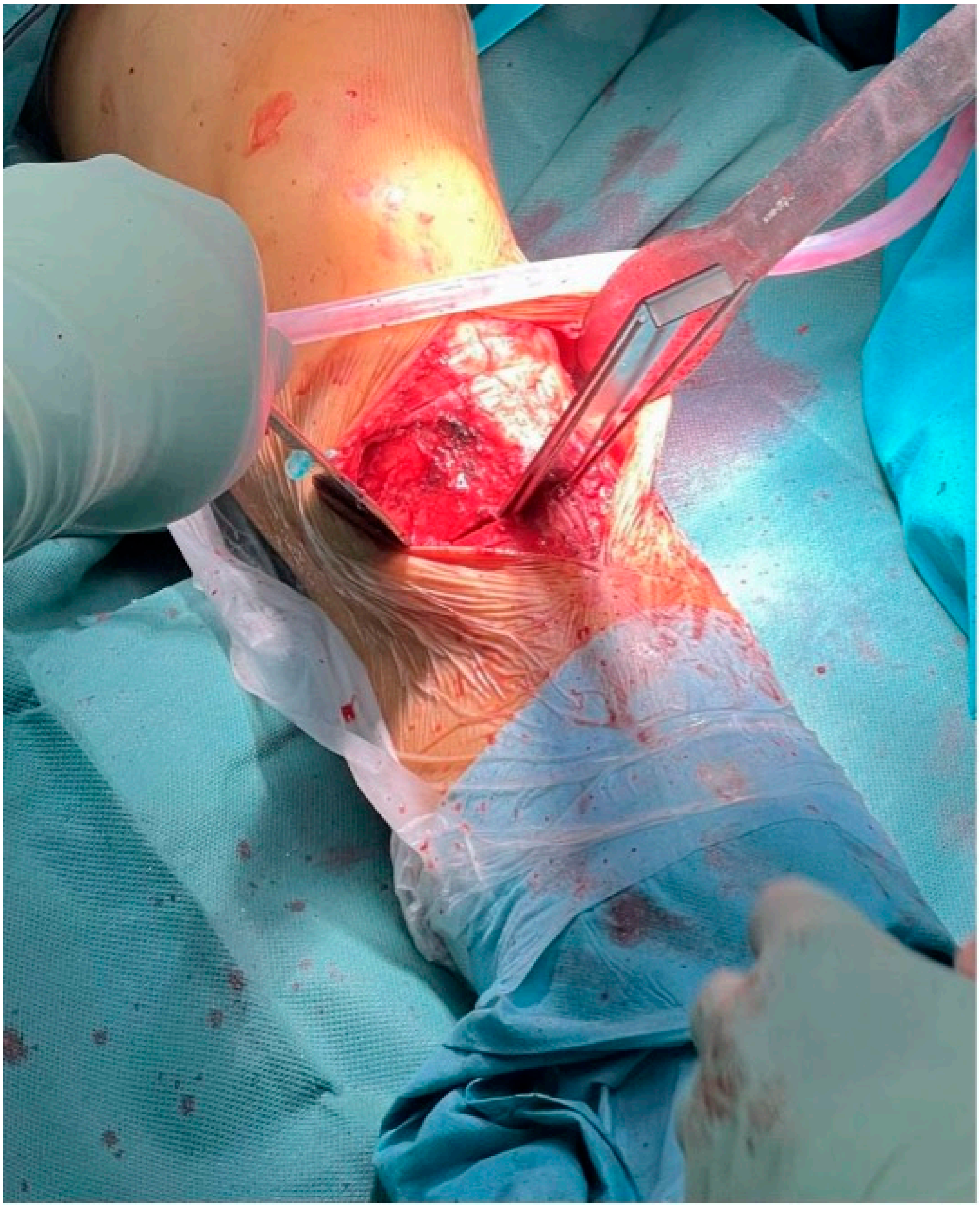

Figure S4. Intraoperative K-wire placement defining the osteotomy orientation and planned correction plane. Fluoroscopic verification of wire trajectory is essential before bone cutting to maintain the intended posterior hinge.. Reprinted from Ref.[11]

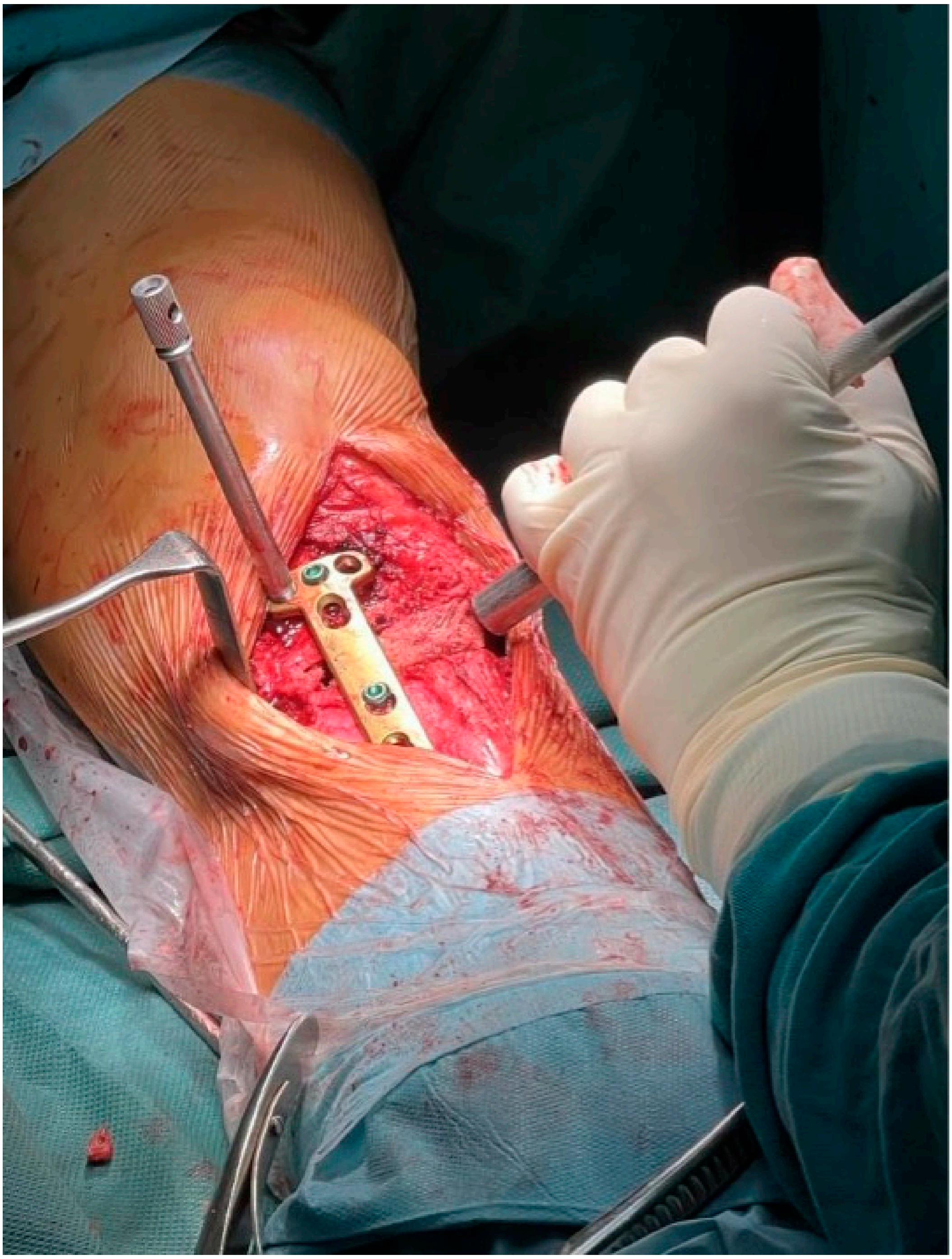

Figure S5. Stable fixation using a TomoFix locking plate following completion of the opening-wedge osteotomy and graft insertion.. Reprinted from Ref.[11]

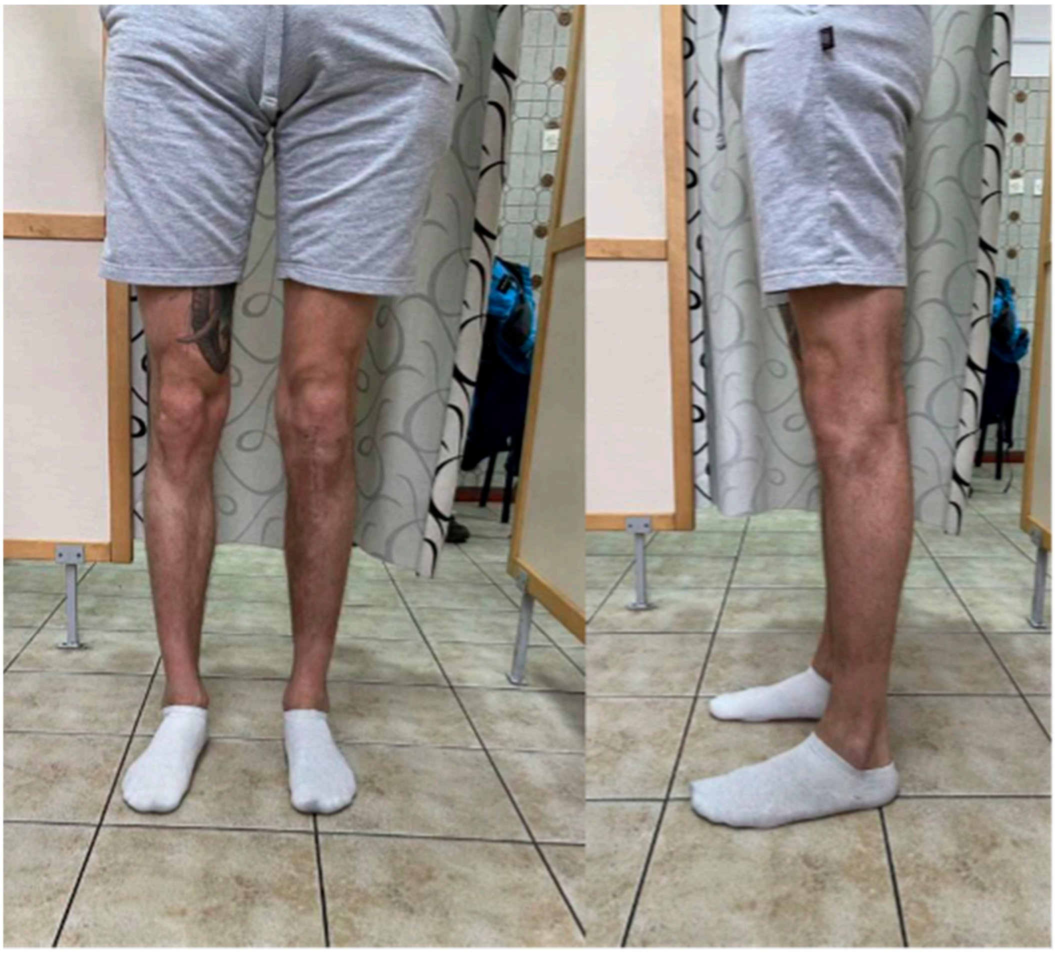

Figure S6. Postoperative clinical correction demonstrating restoration of normal sagittal alignment.. Reprinted from Ref.[11]

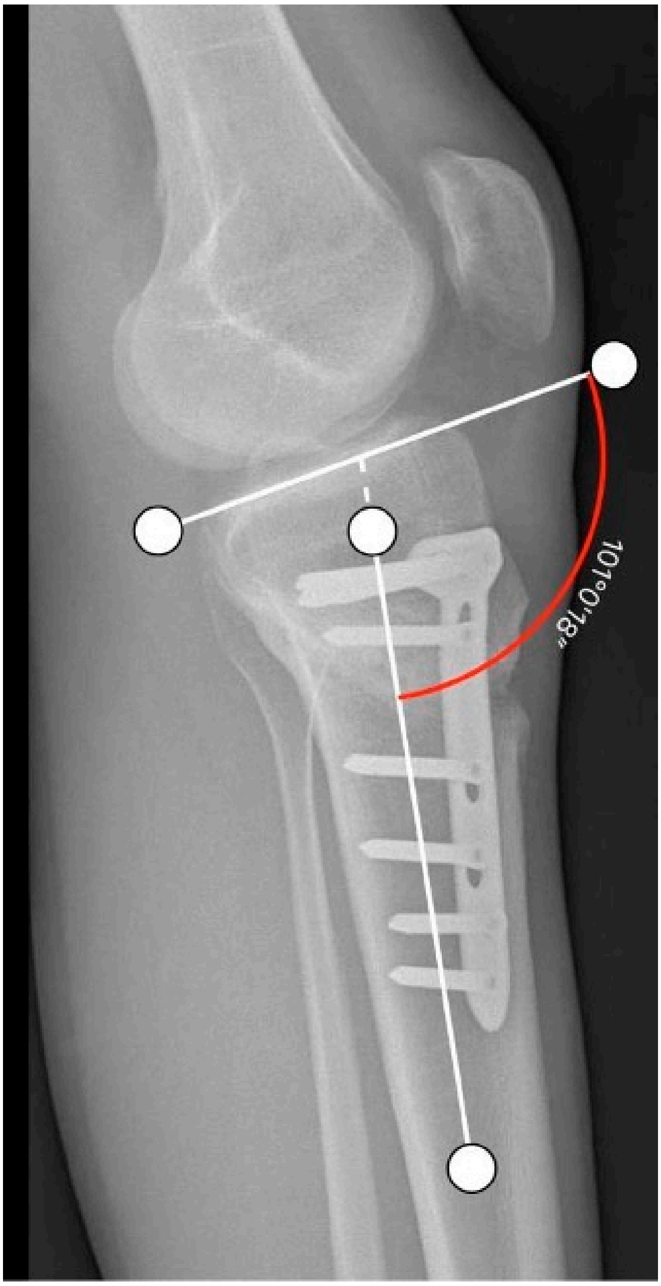

Figure S7. Postoperative lateral radiograph demonstrating restoration of physiological posterior tibial slope to approximately 11° and maintenance of sagittal alignment. Reprinted from Ref.[11]

Table S1. Representative clinical and technical literature on symptomatic genu recurvatum.

**Author**	**Year**	**Design**	**Main pathology**	**Technique or focus**	**Main contribution**
Gill [34]	1931	Historical report	Paralytic recurvatum	Operative correction	Early surgical description
Heyman [35]	1947	Historical report	Paralytic recurvatum	Operative treatment	Early operative experience

Author	Year	Design	Main pathology	Technique or focus	Main contribution
Perry et al. [21]	1976	Clinical report	Paralytic recurvatum	Triple tenodesis	Soft-tissue correction concept
Lecuire et al. [4]	1980	Clinical series	Mixed recurvatum	Operative treatment	Foundational treatment series
Bowen [42]	1983	Technical report	Pediatric recurvatum	Closing-wedge/anterior displacement osteotomy	Alternative osteotomy technique
Mehta and Mukherjee [20]	1991	Clinical series	Poliomyelitis	Femoral flexion osteotomy	Improved ambulation in paralytic deformity
Moroni et al. [5]	1992	Clinical series	Adult osseous recurvatum	Proximal tibial osteotomy	Established adult tibial osteotomy concept
Choi et al. [6]	1999	Clinical series	Growth arrest/post-traumatic	Ilizarov correction	Gradual correction of proximal tibial recurvatum
Dejour et al. [3]	2000	Technical review	Osseous/soft-tissue/combined	Tibial antirecurvatum osteotomy	Etiology-based classification and planning
Piriou et al. [38]	2002	Clinical report	Ligament-related recurvatum	Soft-tissue technique	Ligament-focused correction
Chen et al. [29]	2004	Case report	Physeal growth arrest	Opening wedge and dual plate fixation	Severe osseous recurvatum correction
Van Raaij and de Waal Malefijt [7]	2006	Case series	Idiopathic hyperextension	Anterior opening-wedge PTO	Symptom relief and slope restoration
Babu et al. [43]	2012	Clinical series	Pediatric proximal tibial recurvatum	Ilizarov correction	Gradual correction and pediatric planning
Gaskill et al. [22]	2014	Case report	Recurvatum with valgus	Anterolateral opening-wedge osteotomy	Combined sagittal and coronal correction
Kim et al. [8]	2017	Clinical series	Osseous recurvatum	Anterior open-wedge oblique osteotomy	Modern oblique slope-correction technique
Dean et al. [2]	2020	Systematic review	Symptomatic recurvatum	Multiple surgical techniques	Summarized limited evidence and supported slope-increasing PTO
Rodriguez et al. [23]	2021	Technical note	Posterior instability and recurvatum	Opening-wedge proximal tibial osteotomy	Technique for increased anterior tibial slope
Marques et al. [24]	2022	Technical note	Traumatic recurvatum	Anterior slope correction-flexion osteotomy	Technical approach to traumatic deformity
Bueno et al. [25]	2025	Technical note	Osseous recurvatum with cartilage lesion	Opening-wedge PTO with OATS	Combined slope correction and cartilage restoration
Jawanda et al. [26]	2023	Technical note	Post-traumatic/soft-tissue laxity	Anterior opening-wedge HTO	Contemporary technical approach and perioperative planning
Reddy et al. [33]	2024	Technical note	Sagittal + coronal malalignment; failed PCL reconstruction	Anterior open-wedge osteotomy	Simultaneous sagittal and coronal correction
Georgiadis et al. [32]	2024	Narrative/technical review	Proximal tibial recurvatum-valgus	Multiplanar deformity analysis and corrective options	Emphasized true-plane deformity analysis and combined osseous/ligament evaluation

Author	Year	Design	Main pathology	Technique or focus	Main contribution
Chou et al. [9]	2025	Case series; Level IV	Symptomatic genu recurvatum	Tuberosity-sparing anterior opening-wedge tibial osteotomy	30-patient series: improved recurvatum angle and KOOS; preserved coronal alignment and patellar height
Cohen et al. [10]	2026	Narrative review	PCL insufficiency and genu recurvatum	Anterior opening-wedge HTO / slope restoration	Contemporary synthesis of sagittal geometry, PCL biomechanics, indications, planning, and complications

Table S2. Clinical pearls and pitfalls.

Clinical pearl	Technical rationale	Pitfall / consequence
Define the dominant etiology before selecting treatment	Distinguishes osseous, soft-tissue, neuromuscular, and combined recurvatum	Treating hyperextension alone may leave the primary pathology uncorrected
Measure PTS with a reproducible method on a true lateral radiograph	Allows reliable preoperative planning and postoperative comparison	Projection error or inconsistent axis definition may create false correction values
Assess cruciate and posterolateral structures with MRI	Confirms whether osteotomy alone is appropriate	Unrecognized ligament insufficiency may cause persistent instability
Use CT-based planning when proximal tibial morphology is complex	Improves understanding of osteotomy level, wedge geometry, and multiplanar deformity	Incorrect osteotomy level or wedge size may compromise correction
Verify K-wire trajectory fluoroscopically before cutting	Defines the intended osteotomy plane and protects the planned hinge location	Malposition may produce an unintended osteotomy plane
Complete the posterior osteotomy progressively under fluoroscopy	Reduces uncontrolled stress concentration at the posterior cortex	Blind or incomplete posterior cutting followed by forceful opening may fracture the hinge
Preserve a posterior cortical bridge	Maintains a stable center of rotation during gradual slope correction	Posterior hinge fracture may destabilize the osteotomy and cause loss of correction control
Open the wedge gradually in small increments	Permits repeated assessment of hinge integrity and sagittal correction	Rapid or asymmetric opening increases hinge stress and risks overcorrection
Confirm the planned correction before definitive fixation	Balances elimination of recurvatum against excessive PTS	Undercorrection leaves residual hyperextension; overcorrection may alter cruciate biomechanics
Support the gap and use stable locking-plate fixation	Maintains correction during osteotomy healing	Inadequate support may contribute to correction loss, delayed union, or hardware failure
Use structured rehabilitation with radiographic progression of loading	Protects the osteotomy while restoring motion and strength	Premature loading or delayed rehabilitation may impair healing or functional recovery
